# Diagnostic testing for SARS-CoV-2 infection in HHT patients: nasopharyngeal versus oropharyngeal swab

**DOI:** 10.1186/s13023-020-01628-w

**Published:** 2020-12-18

**Authors:** Fabio Pagella, Roberta Lizzio, Sara Ugolini, Giuseppe Spinozzi, Eugenia Maiorano, Patrizia Suppressa, Carlo Sabbà, Elina Matti

**Affiliations:** 1 Department of Otorhinolaryngology, Fondazione IRCCS Policlinico San Matteo, Pavia, Italy; 2 Department of Otorhinolaryngology, University of Pavia, Pavia, Italy; 3 Department of Internal Medicine and Rare Disease Centre “C. Frugoni” University Hospital of Bari, Bari, Italy

**Keywords:** Nasopharyngeal swab, Oropharyngeal swab, SARS-CoV-2, COVID-19, RT-PCR, SARS-CoV-2 diagnosis, HHT, Nosebleeding, Epistaxis

## Abstract

On March 11, 2020, WHO has defined the novel coronavirus disease SARS-CoV-2 (COVID-19) outbreak as a pandemic that still today continues to affect much of the world. Among the reasons for the rapid spread of SARS-CoV-2 infection, there is the role of asymptomatic or minimally symptomatic carriers. Therefore diagnostic testing is central to contain the global pandemic. Up to now real-time reverse transcriptase polymerase chain reaction-based molecular assays for detecting SARS-CoV-2 in respiratory specimens is the current reference standard for COVID-19 diagnosis. Based on current knowledge regarding the sensitivity of the molecular test, the highest positive detection rate is from lower respiratory tract specimens; alternatively it is possible to perform a nasopharyngeal or oropharyngeal swab. Nasopharyngeal swab is the preferred choice for SARS-CoV-2 testing since it seems to have a greater sensitivity; however the procedure is not always free of complications and an epistaxis can occur. Among patients with greatest risk of massive nosebleed there are HHT patients. Hereditary hemorrhagic telangiectasia is an autosomal dominant disease that leads to multiregional mucocutanous telangiectases and visceral arteriovenous malformations. Clinically, the presence of telangiectases in nasal mucosa is the cause of recurrent epistaxis. In HHT patients the execution of the nasopharyngeal swab can determine from little or no consequences to a massive epistaxis leading to the necessity of nasal packing generally followed by hospital admission. In HHT patients undergoing a diagnostic test to evaluate the SARS-CoV-2 infection status, especially in those patients with frequent epistaxis with a history of anemia and repeated hospitalizations, it is therefore advisable to perform an oropharyngeal swab. This, compared to the nasopharyngeal swab, exposes to a lower risk of severe nosebleeds related treatments, such as blood transfusions or invasive procedures. According to the risk-benefit assessment and based on our experience, we consider that, despite a lower diagnostic sensitivity, oropharyngeal swab is preferable to nasopharyngeal swab for the diagnosis of SARS CoV-2 infection in patients with HHT.

Dear Editor,

Since the World Health Organization (WHO) declared the novel Coronavirus disease (COVID-19) outbreak as pandemic, on March 11th, 2020, the virus has spread to more than 48,786,440 individuals, resulting in 1,234,839 confirmed deaths [1].

Among the reasons for this rapid spread of SARS-CoV-2 infection, there is not only the high transmissibility of the virus, but also the role of asymptomatic or minimally symptomatic carriers. Therefore diagnostic testing, to identify people infected with COVID-19, is central to contain the global pandemic, implementing the strict isolation recommended by all governments [2].

Up to now real-time reverse transcriptase polymerase chain reaction (RT-PCR)-based molecular assays for detecting SARS-CoV-2 in respiratory specimens are the current reference standard for COVID-19 diagnosis [2].

Based on current knowledge regarding the sensitivity of the molecular test, the highest positive detection rate is from lower respiratory tract specimens with a sensitivity from 93 to 100% [3, 4]. However, especially in asymptomatic or paucisymptomatic patients, collection of these specimens is not easily feasible; alternatively it is possible to perform a nasopharyngeal or oropharyngeal swab. Between the two sampling sites a greater sensitivity is recorded in the nasopharyngeal sample, described around 63–73%, while the oropharyngeal swab has a sensitivity ranging from 32 to 60% [3, 4].

Nasopharyngeal swabs are considered easy and quick to collect, they are not time-consuming and they can be performed anywhere [5]. However during the diagnostic procedure, especially if not performed correctly, an epistaxis can occur. To reduce this risk, it is recommended to provide training for the health personnel that is going to collect the samples, to inform the patient about the procedure and ask for previous nasal trauma or surgery, septal deviations, history of recurrent nasal bleeding or known coagulopathy [6].

Among patients with greatest risk of massive nosebleed there are HHT patients. Hereditary hemorrhagic telangiectasia also known as Rendu-Osler-Weber disease, is an autosomal dominant disease and its prevalence is approximately 1/6000 [7]. HHT has an almost complete age-related penetrance and leads to multiregional mucocutanous telangiectases and visceral arteriovenous malformations [8]. Clinically, the presence of telangiectases in nasal mucosa leads to recurrent epistaxis that affect up to 96% of patients [9]. There is considerable variability from patients whose nosebleeds are limited to a few blood spots on a handkerchief to patients in whom nosebleed severity can lead to chronic anemia and may require a large number of blood transfusions, thus resulting in a worse quality of life [10].

During the execution of a diagnostic nasopharyngeal swab for Covid-19, a massive epistaxis could be triggered in HHT patients, leading to the necessity of nasal packing generally followed by hospital admission. This represents a pejorative factor, because placement and/or removal of nasal packing can lead to mucosal trauma and additional bleeding. For these reasons acute epistaxis HHT-related should be managed with atraumatic packing like resorbable packing materials to prevent rebleeding and to avoid the need for more invasive interventions [11]. However, despite an increased risk of nosebleed, this is not predictable, and also in HHT patients suffering from minor self-managed epistaxis, the execution of the nasopharyngeal swab can determine from little or no consequences to a massive epistaxis difficult to manage.

In HHT patients undergoing a diagnostic test to evaluate the SARS-CoV-2 infection status, especially in those patients with frequent epistaxis with a history of mild to severe anemia and repeated hospitalizations, it is therefore advisable, in our opinion, to perform an oropharyngeal swab.

This, compared to the nasopharyngeal swab, exposes to a lower risk of severe nosebleeds related treatments, such as blood transfusions or invasive procedures ranging from prolonged nasal packing to endovascular embolization [12].

It is also advisable, especially in patients with a clinical situation strongly suggestive for COVID-19, to perform a second oropharyngeal swab, in order to increase the diagnostic sensitivity.

According to the risk-benefit assessment and based on our experience, we consider that, despite a lower diagnostic sensitivity, oropharyngeal swab is preferable to nasopharyngeal swab for the diagnosis of SARS CoV-2 infection in patients with HHT.

## Data Availability

No analysis of clinical data was conducted.

## Abbreviations

WHO: World Health Organization
COVID 19: Coronavirus disease 2019
SARS-CoV-2: Severe acute respiratory syndrome coronavirus 2
RT-PCR: Reverse transcriptase polymerase chain reaction
HHT: Hereditary hemorrhagic telangiectasia

## References

[1] World Health Organization. Coronavirus disease 2019 (COVID 2019) situation report—90, https://www.who.int/docs/default-source/coronaviruse/situation-reports/20200419-sitrep-90-covid-19.pdf?sfvrsn=551d47fd_4. Accessed 07 Nov 2020.

[2] Cheng MP, Papenburg J, Desjardins M, Kanjilal S, Quach C, Libman M, Dittrich S, Yansouni CP (2020). Diagnostic testing for severe acute respiratory syndrome-related coronavirus-2: a narrative review. Ann Intern Med.

[3] Yang Y, Yang M, Shen C, Wang F, Yuan J, Li J (2020). Evaluating the accuracy of different respiratory specimens in the laboratory diagnosis and monitoring the viral shedding of 2019-nCoV infections. MedRxiv.

[4] Wang W, Xu Y, Gao R (2020). Detection of SARS-CoV-2 in different types of clinical specimens. JAMA.

[5] Tagliabue M, Pietrobon G, Ugolini S, Chu F, Ansarin M (2020). Nasopharyngeal swabs during SARS-CoV-2 pandemic: a role for the otolaryngologist. Eur Arch Otorhinolaryngol.

[6] Coden E, Russo F, Arosio AD, Castelnuovo P, Karligkiotis A, Volpi L (2020). Optimum naso-oropharyngeal swab procedure for COVID-19: step by step preparation and technical hints. Laryngoscope.

[7] Orphanet. The portal of rare diseases and orphan drugs. Hereditary hemorrhagic telangiectasia. https://www.orpha.net/consor/cgi-bin/OC_Exp.php?Expert=774&lng=EN.

[8] Shovlin CL, Guttmacher AE, Buscarini E, Faughnan ME, Hyland RH, Westermann CJ, Kjeldsen AD, Plauchu H (2000). Diagnostic criteria for hereditary hemorrhagic telangiectasia (Rendu-Osler-weber syndrome). Am J Med Genet.

[9] Sadick H, Sadick M, Gotte K, Naim R, Riedel F, Bran G, Hörmann K (2006). Hereditary hemorrhagic telangiectasia: An update on clinical manifestations and diagnostic measures. Wien Klin Wochenschr.

[10] Pagella F, Matti E, Chu F, Pusateri A, Tinelli C (2013). Argon plasma coagulation is an effective treatment for hereditary hemorrhagic telangiectasia patients with severe nosebleeds. Acta Otolaryngol.

[11] Tunkel DE, Anne S, Payne SC (2020). Clinical practice guideline: nosebleed (epistaxis). Otolaryngol Head Neck Surg.

[12] Faughnan ME, Palda VA, Garcia-Tsao G, Geisthoff UW, McDonald J (2011). International guidelines for the diagnosis and management of hereditary haemorrhagic telangiectasia. J Med Genet.

